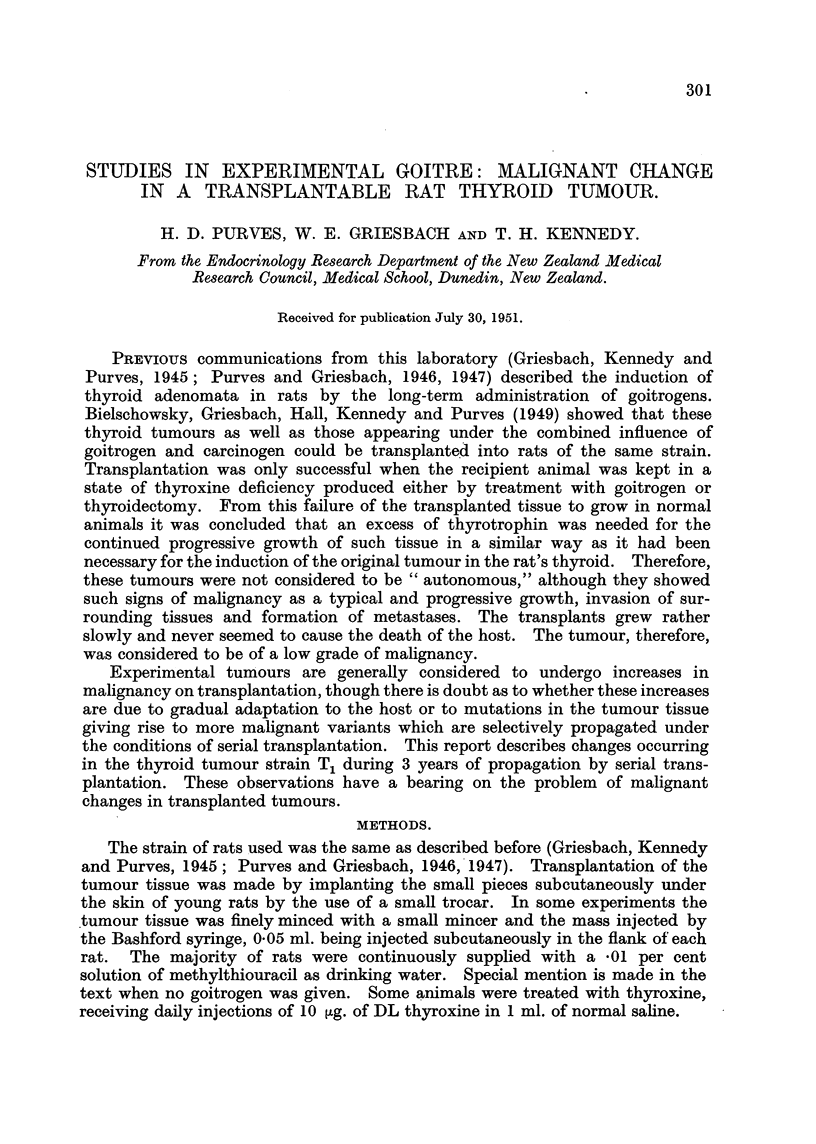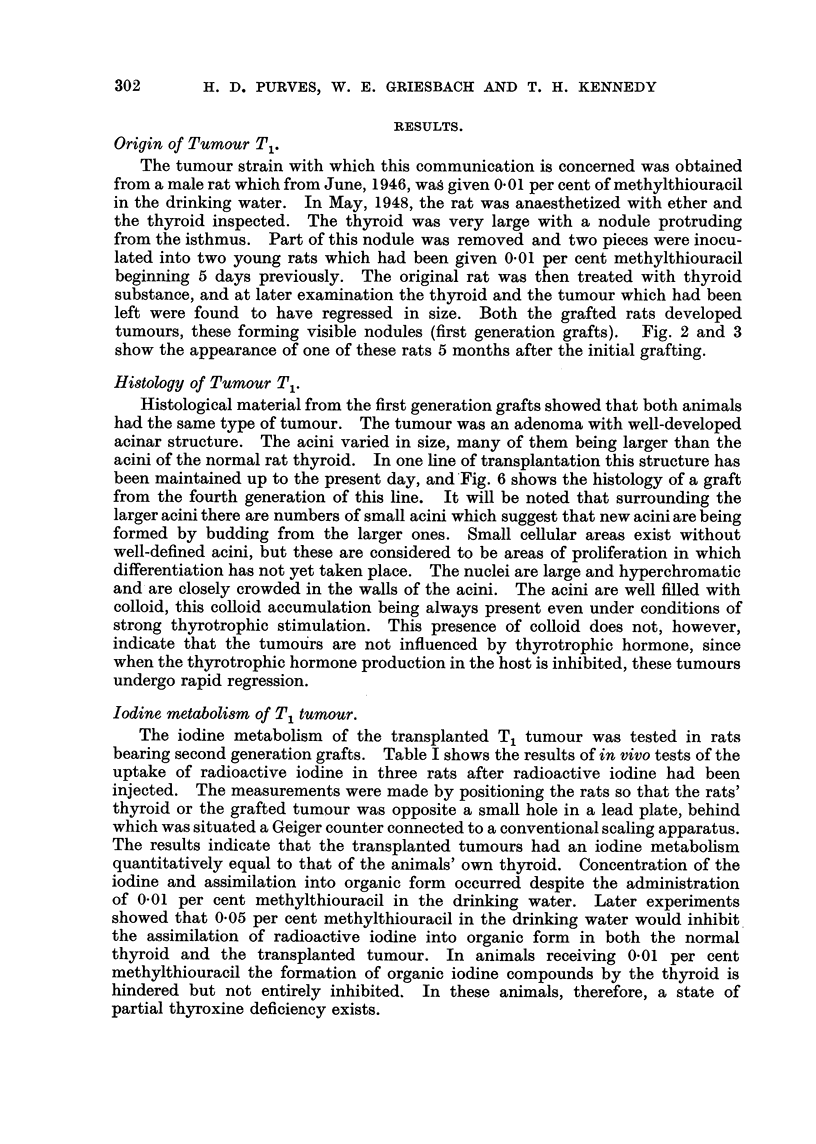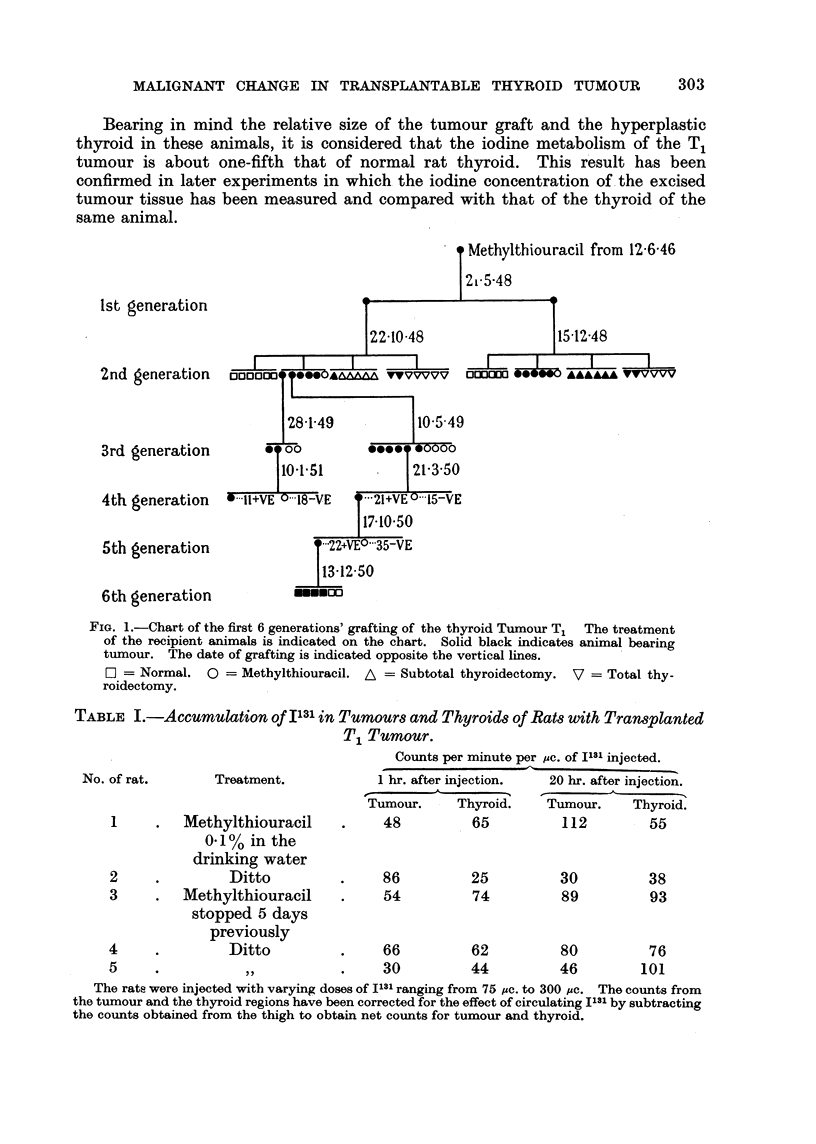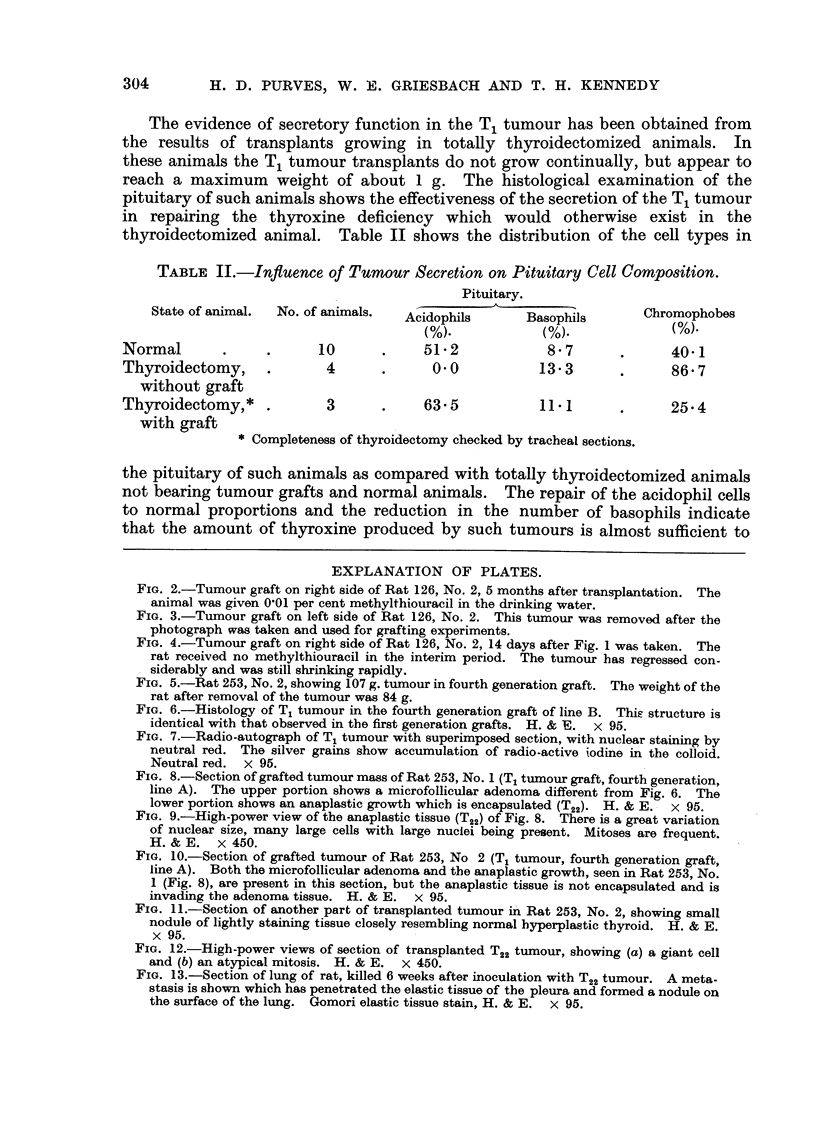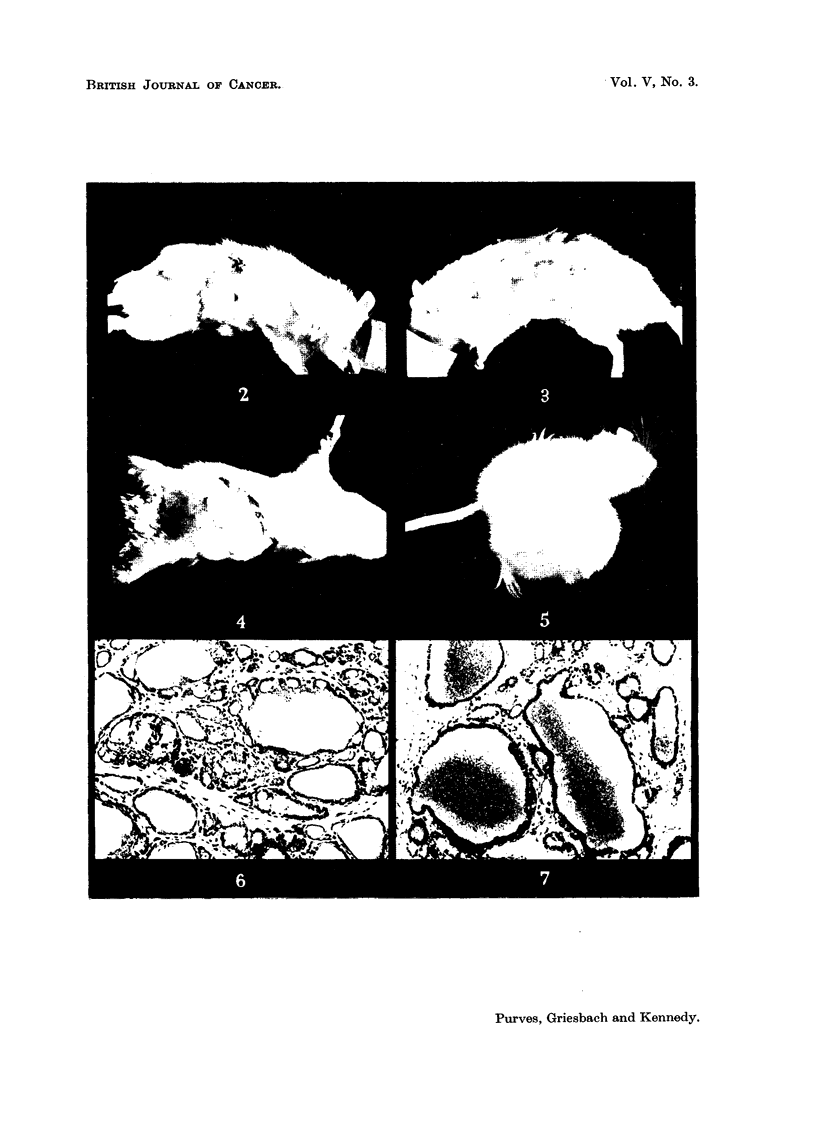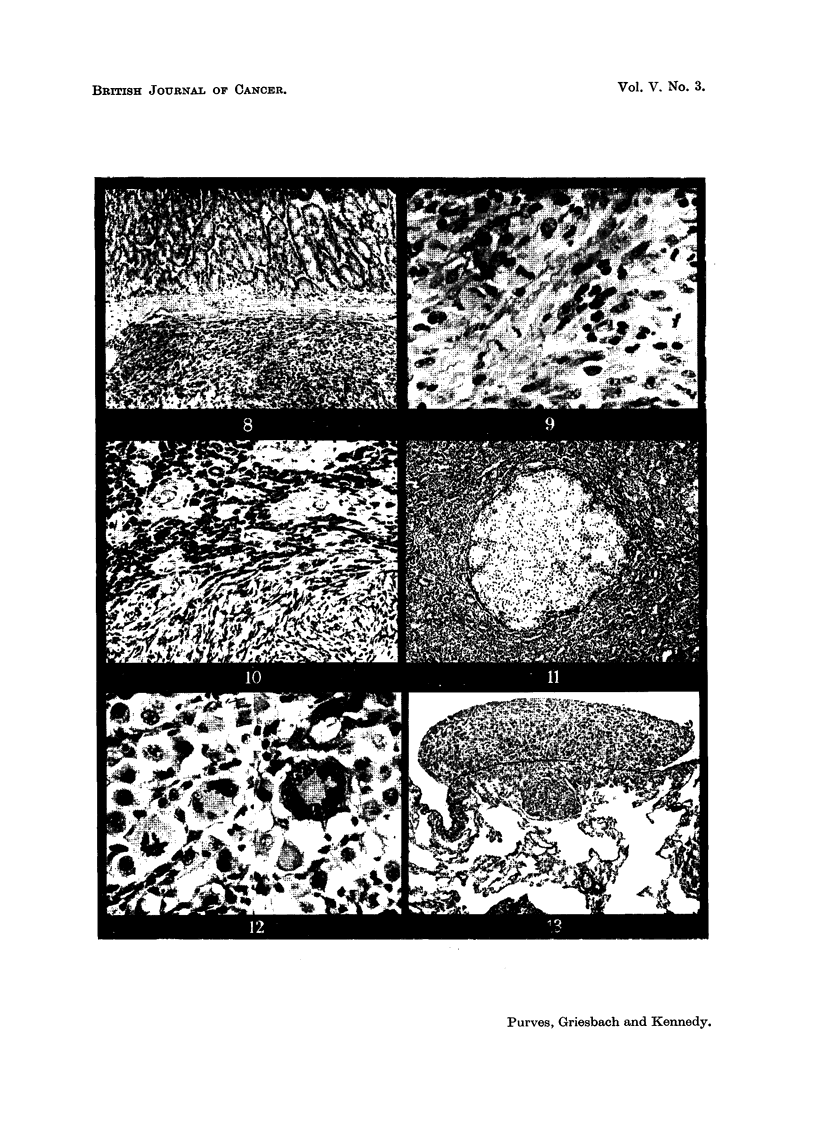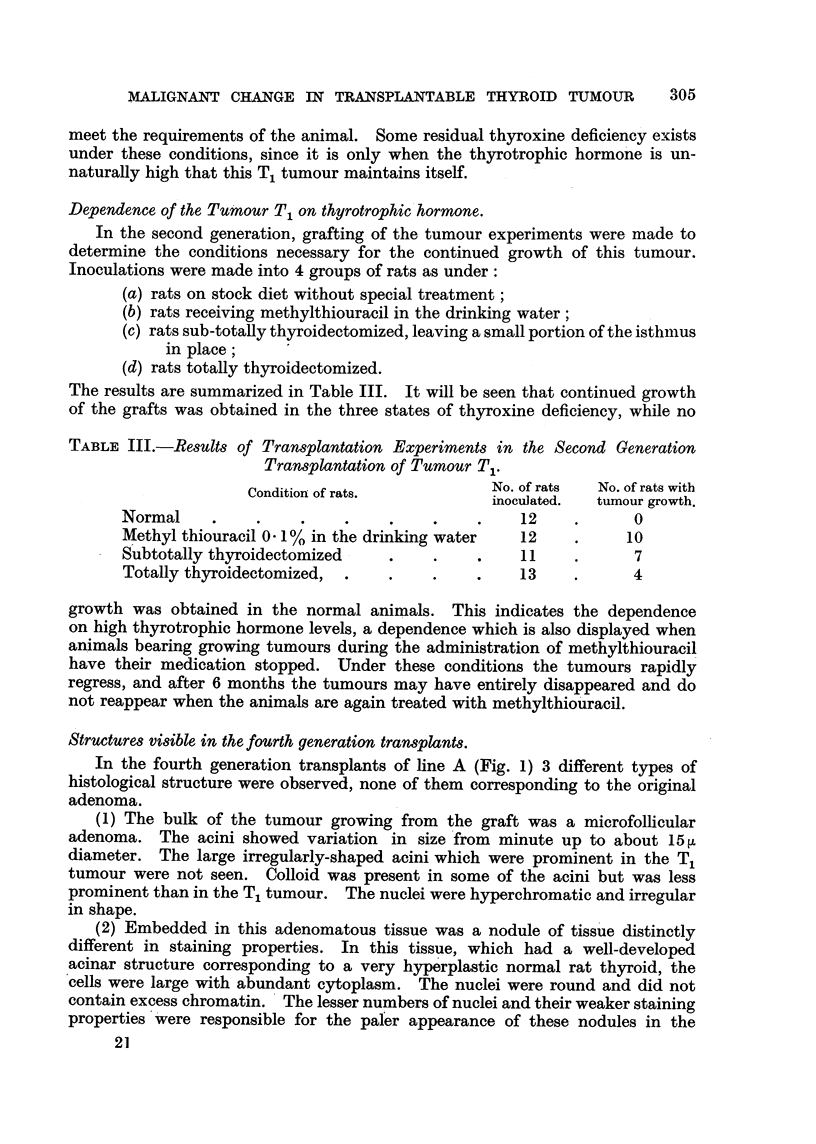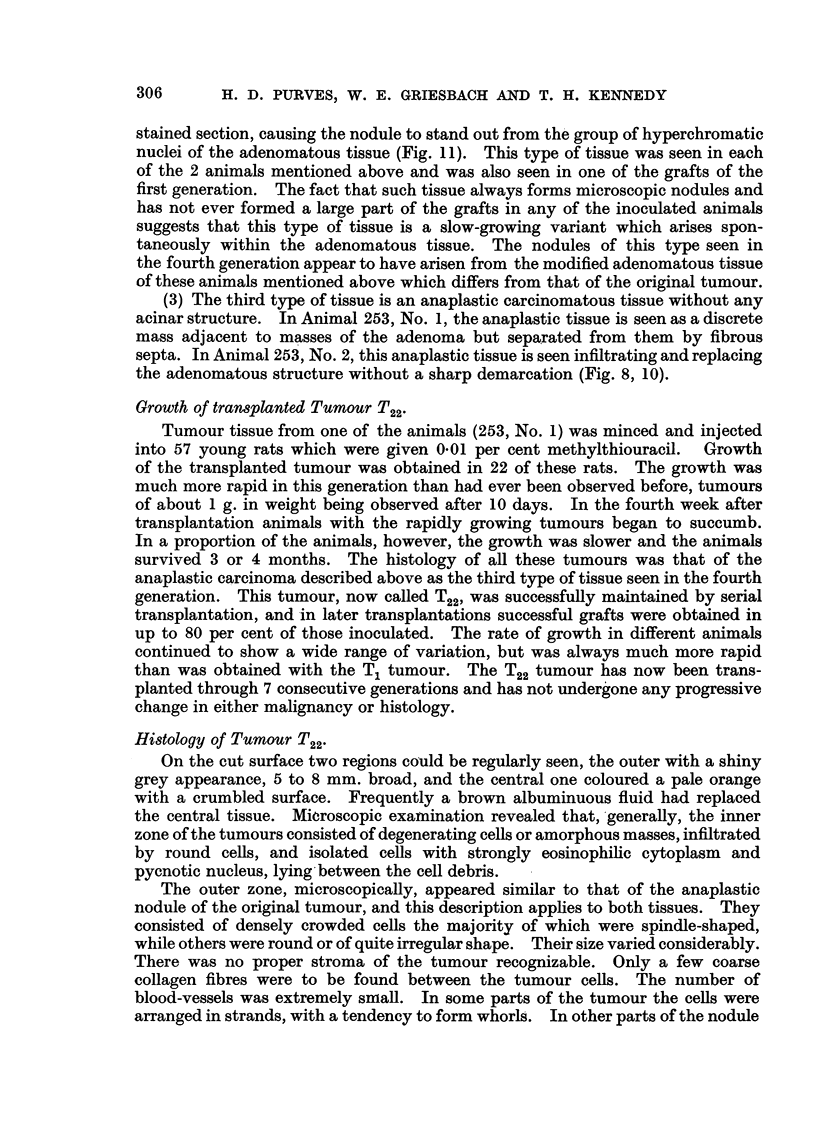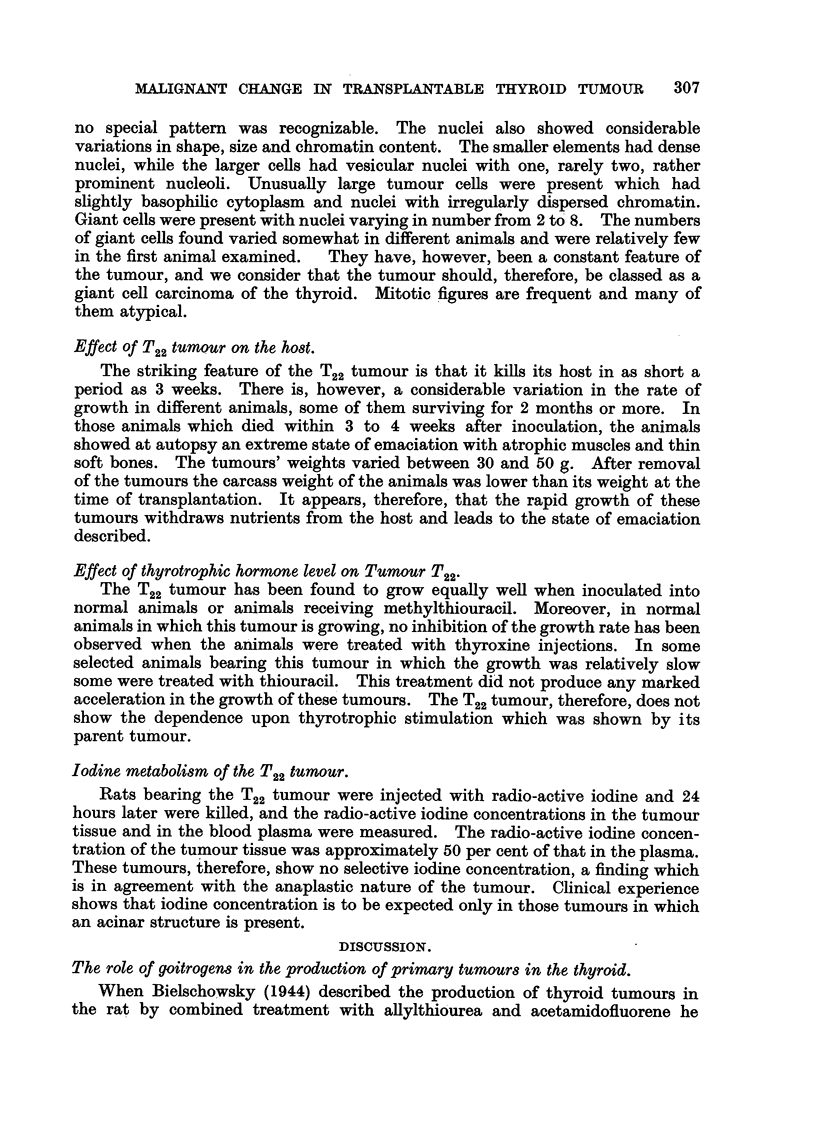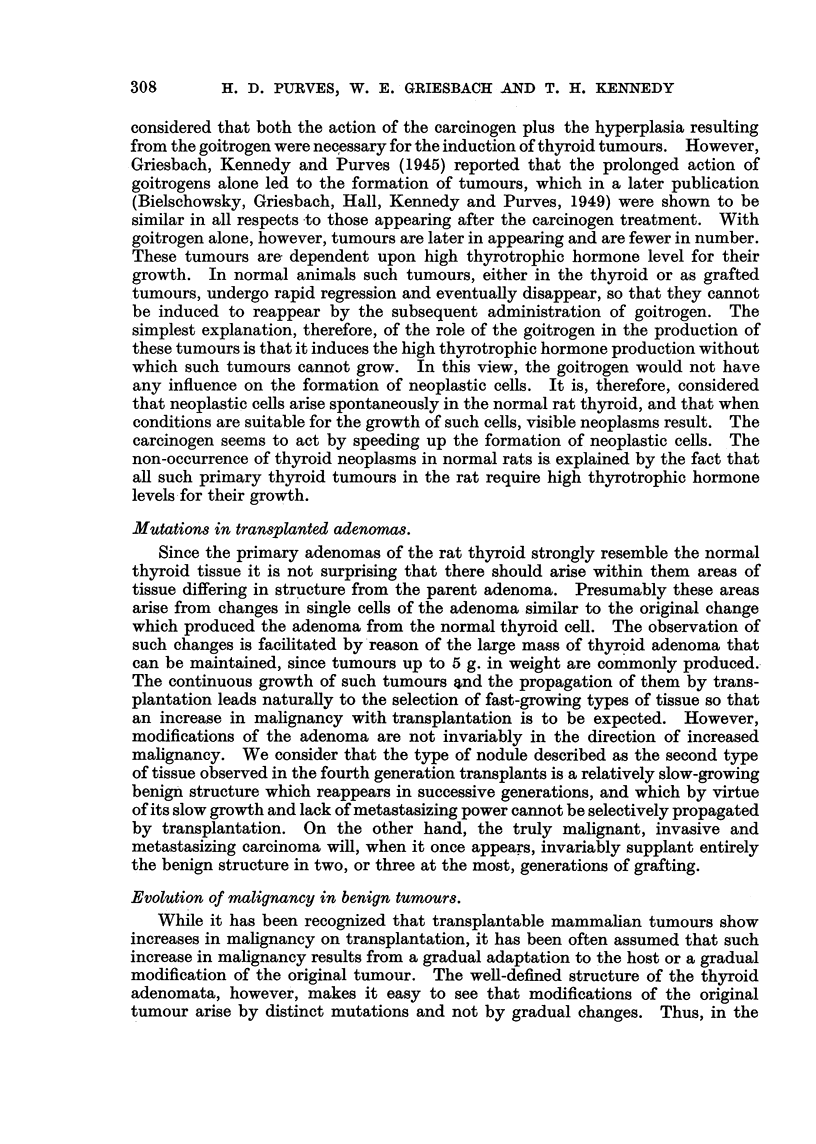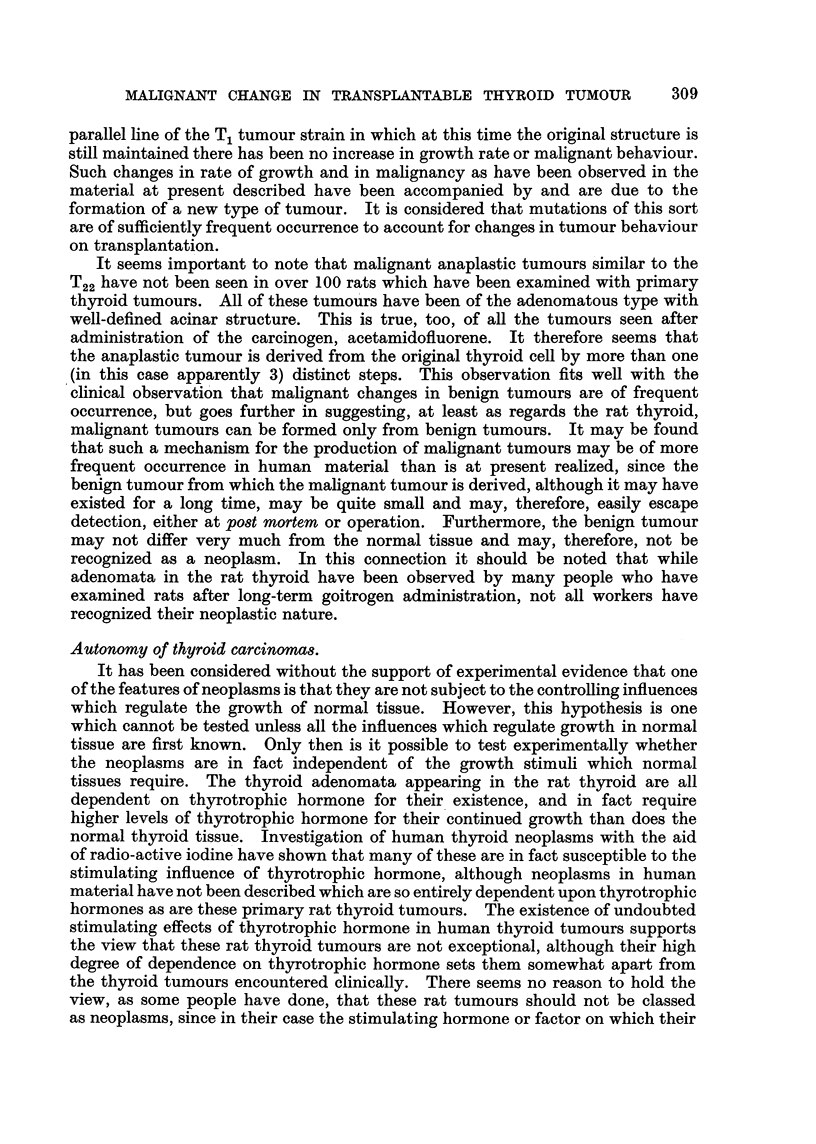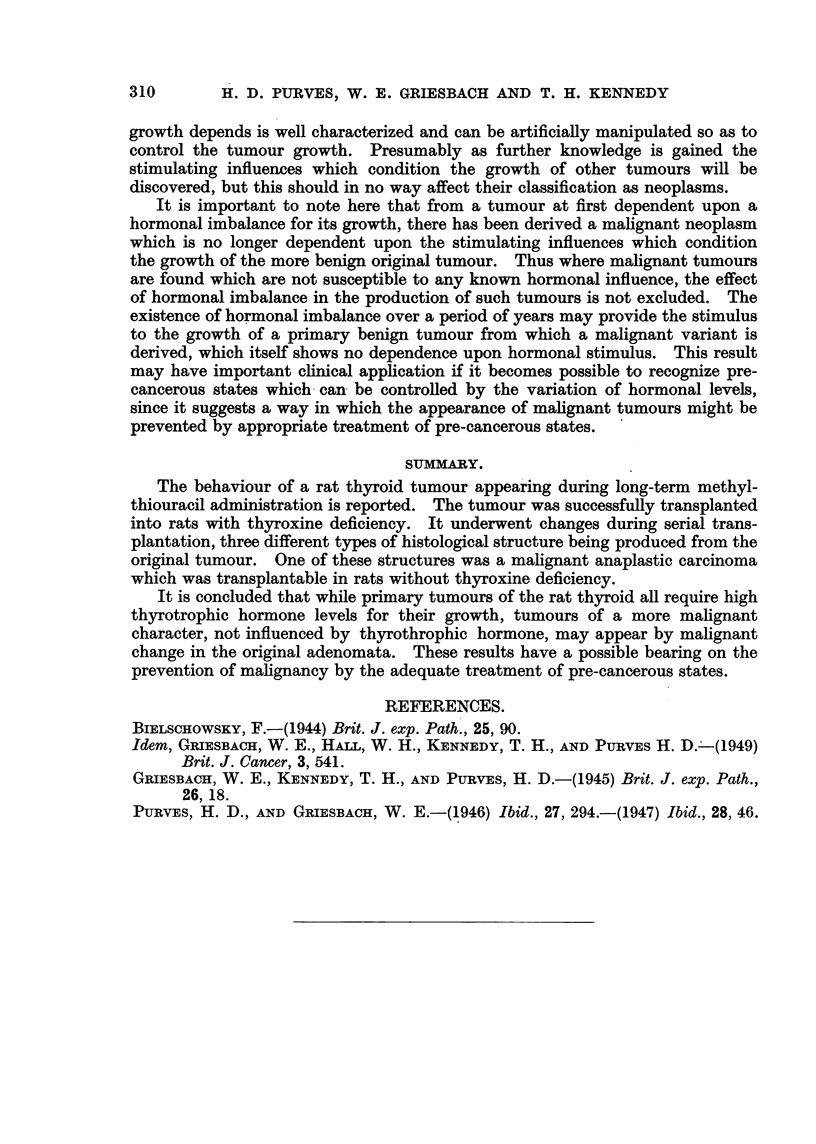# Studies in Experimental Goitre: Malignant Change in a Transplantable Rat Thyroid Tumour

**DOI:** 10.1038/bjc.1951.31

**Published:** 1951-09

**Authors:** H. D. Purves, W. E. Griesbach, T. H. Kennedy

## Abstract

**Images:**


### STUDIES IN EXPERIMENTAL GOITRE: MALIGNANT CHANGE IN A TRANSPLANTABLE RAT THYROID TUMOUR.

H. D. PURVES, W. E. GRIESBACH AND T. H. KENNEDY.

*From the Endocrinology Research Department of the New Zealand Medical Research Council, Medical School, Dunedin, New Zealand.*

Received for publication July 30, 1951.

PREVIOUS communications from this laboratory (Griesbach, Kennedy and Purves, 1945; Purves and Griesbach, 1946, 1947) described the induction of thyroid adenomata in rats by the long-term administration of goitrogens. Bielschowsky, Griesbach, Hall, Kennedy and Purves (1949) showed that these thyroid tumours as well as those appearing under the combined influence of goitrogen and carcinogen could be transplanted into rats of the same strain. Transplantation was only successful when the recipient animal was kept in a state of thyroxine deficiency produced either by treatment with goitrogen or thyroidectomy. From this failure of the transplanted tissue to grow in normal animals it was concluded that an excess of thyrotrophin was needed for the continued progressive growth of such tissue in a similar way as it had been necessary for the induction of the original tumour in the rat's thyroid. Therefore, these tumours were not considered to be "autonomous," although they showed such signs of malignancy as a typical and progressive growth, invasion of surrounding tissues and formation of metastases. The transplants grew rather slowly and never seemed to cause the death of the host. The tumour, therefore, was considered to be of a low grade of malignancy.

Experimental tumours are generally considered to undergo increases in malignancy on transplantation, though there is doubt as to whether these increases are due to gradual adaptation to the host or to mutations in the tumour tissue giving rise to more malignant variants which are selectively propagated under the conditions of serial transplantation. This report describes changes occurring in the thyroid tumour strain $T_1$ during 3 years of propagation by serial transplantation. These observations have a bearing on the problem of malignant changes in transplanted tumours.

### METHODS.

The strain of rats used was the same as described before (Griesbach, Kennedy and Purves, 1945; Purves and Griesbach, 1946, 1947). Transplantation of the tumour tissue was made by implanting the small pieces subcutaneously under the skin of young rats by the use of a small trocar. In some experiments the tumour tissue was finely minced with a small mincer and the mass injected by the Bashford syringe, 0·05 ml. being injected subcutaneously in the flank of each rat. The majority of rats were continuously supplied with a ·01 per cent solution of methylthiouracil as drinking water. Special mention is made in the text when no goitrogen was given. Some animals were treated with thyroxine, receiving daily injections of 10 μg. of DL thyroxine in 1 ml. of normal saline.

RESULTS.

*Origin of Tumour $T_1$.*

The tumour strain with which this communication is concerned was obtained from a male rat which from June, 1946, was given 0·01 per cent of methylthiouracil in the drinking water. In May, 1948, the rat was anaesthetized with ether and the thyroid inspected. The thyroid was very large with a nodule protruding from the isthmus. Part of this nodule was removed and two pieces were inoculated into two young rats which had been given 0·01 per cent methylthiouracil beginning 5 days previously. The original rat was then treated with thyroid substance, and at later examination the thyroid and the tumour which had been left were found to have regressed in size. Both the grafted rats developed tumours, these forming visible nodules (first generation grafts). Fig. 2 and 3 show the appearance of one of these rats 5 months after the initial grafting.

*Histology of Tumour $T_1$.*

Histological material from the first generation grafts showed that both animals had the same type of tumour. The tumour was an adenoma with well-developed acinar structure. The acini varied in size, many of them being larger than the acini of the normal rat thyroid. In one line of transplantation this structure has been maintained up to the present day, and Fig. 6 shows the histology of a graft from the fourth generation of this line. It will be noted that surrounding the larger acini there are numbers of small acini which suggest that new acini are being formed by budding from the larger ones. Small cellular areas exist without well-defined acini, but these are considered to be areas of proliferation in which differentiation has not yet taken place. The nuclei are large and hyperchromatic and are closely crowded in the walls of the acini. The acini are well filled with colloid, this colloid accumulation being always present even under conditions of strong thyrotrophic stimulation. This presence of colloid does not, however, indicate that the tumours are not influenced by thyrotrophic hormone, since when the thyrotrophic hormone production in the host is inhibited, these tumours undergo rapid regression.

*Iodine metabolism of $T_1$ tumour.*

The iodine metabolism of the transplanted $T_1$ tumour was tested in rats bearing second generation grafts. Table I shows the results of *in vivo* tests of the uptake of radioactive iodine in three rats after radioactive iodine had been injected. The measurements were made by positioning the rats so that the rats' thyroid or the grafted tumour was opposite a small hole in a lead plate, behind which was situated a Geiger counter connected to a conventional scaling apparatus. The results indicate that the transplanted tumours had an iodine metabolism quantitatively equal to that of the animals' own thyroid. Concentration of the iodine and assimilation into organic form occurred despite the administration of 0·01 per cent methylthiouracil in the drinking water. Later experiments showed that 0·05 per cent methylthiouracil in the drinking water would inhibit the assimilation of radioactive iodine into organic form in both the normal thyroid and the transplanted tumour. In animals receiving 0·01 per cent methylthiouracil the formation of organic iodine compounds by the thyroid is hindered but not entirely inhibited. In these animals, therefore, a state of partial thyroxine deficiency exists.

Bearing in mind the relative size of the tumour graft and the hyperplastic thyroid in these animals, it is considered that the iodine metabolism of the $T_1$ tumour is about one-fifth that of normal rat thyroid. This result has been confirmed in later experiments in which the iodine concentration of the excised tumour tissue has been measured and compared with that of the thyroid of the same animal.

FIG. 1.—Chart of the first 6 generations' grafting of the thyroid Tumour $T_1$ The treatment of the recipient animals is indicated on the chart. Solid black indicates animal bearing tumour. The date of grafting is indicated opposite the vertical lines.

□ = Normal. ○ = Methylthiouracil. △ = Subtotal thyroidectomy. ▽ = Total thyroidectomy.

TABLE I.—*Accumulation of* $I^{131}$ *in Tumours and Thyroids of Rats with Transplanted* $T_1$ *Tumour.*

| No. of rat. | Treatment. | Counts per minute per μc. of $I^{131}$ injected. 1 hr. after injection. Tumour. | Thyroid. | 20 hr. after injection. Tumour. | Thyroid. |
|---|---|---|---|---|---|
| 1 | Methylthiouracil 0·1% in the drinking water | 48 | 65 | 112 | 55 |
| 2 | Ditto | 86 | 25 | 30 | 38 |
| 3 | Methylthiouracil stopped 5 days previously | 54 | 74 | 89 | 93 |
| 4 | Ditto | 66 | 62 | 80 | 76 |
| 5 | ,, | 30 | 44 | 46 | 101 |

The rats were injected with varying doses of $I^{131}$ ranging from 75 μc. to 300 μc. The counts from the tumour and the thyroid regions have been corrected for the effect of circulating $I^{131}$ by subtracting the counts obtained from the thigh to obtain net counts for tumour and thyroid.

The evidence of secretory function in the $T_1$ tumour has been obtained from the results of transplants growing in totally thyroidectomized animals. In these animals the $T_1$ tumour transplants do not grow continually, but appear to reach a maximum weight of about 1 g. The histological examination of the pituitary of such animals shows the effectiveness of the secretion of the $T_1$ tumour in repairing the thyroxine deficiency which would otherwise exist in the thyroidectomized animal. Table II shows the distribution of the cell types in

TABLE II.—*Influence of Tumour Secretion on Pituitary Cell Composition.*

| State of animal. | No. of animals. | Pituitary. Acidophils (%). | Basophils (%). | Chromophobes (%). |
|---|---|---|---|---|
| Normal | 10 | 51·2 | 8·7 | 40·1 |
| Thyroidectomy, without graft | 4 | 0·0 | 13·3 | 86·7 |
| Thyroidectomy,* with graft | 3 | 63·5 | 11·1 | 25·4 |

* Completeness of thyroidectomy checked by tracheal sections.

the pituitary of such animals as compared with totally thyroidectomized animals not bearing tumour grafts and normal animals. The repair of the acidophil cells to normal proportions and the reduction in the number of basophils indicate that the amount of thyroxine produced by such tumours is almost sufficient to

EXPLANATION OF PLATES.

FIG. 2.—Tumour graft on right side of Rat 126, No. 2, 5 months after transplantation. The animal was given 0·01 per cent methylthiouracil in the drinking water.

FIG. 3.—Tumour graft on left side of Rat 126, No. 2. This tumour was removed after the photograph was taken and used for grafting experiments.

FIG. 4.—Tumour graft on right side of Rat 126, No. 2, 14 days after Fig. 1 was taken. The rat received no methylthiouracil in the interim period. The tumour has regressed considerably and was still shrinking rapidly.

FIG. 5.—Rat 253, No. 2, showing 107 g. tumour in fourth generation graft. The weight of the rat after removal of the tumour was 84 g.

FIG. 6.—Histology of $T_1$ tumour in the fourth generation graft of line B. This structure is identical with that observed in the first generation grafts. H. & E. × 95.

FIG. 7.—Radio-autograph of $T_1$ tumour with superimposed section, with nuclear staining by neutral red. The silver grains show accumulation of radio-active iodine in the colloid. Neutral red. × 95.

FIG. 8.—Section of grafted tumour mass of Rat 253, No. 1 ($T_1$ tumour graft, fourth generation, line A). The upper portion shows a microfollicular adenoma different from Fig. 6. The lower portion shows an anaplastic growth which is encapsulated ($T_{22}$). H. & E. × 95.

FIG. 9.—High-power view of the anaplastic tissue ($T_{22}$) of Fig. 8. There is a great variation of nuclear size, many large cells with large nuclei being present. Mitoses are frequent. H. & E. × 450.

FIG. 10.—Section of grafted tumour of Rat 253, No 2 ($T_1$ tumour, fourth generation graft, line A). Both the microfollicular adenoma and the anaplastic growth, seen in Rat 253, No. 1 (Fig. 8), are present in this section, but the anaplastic tissue is not encapsulated and is invading the adenoma tissue. H. & E. × 95.

FIG. 11.—Section of another part of transplanted tumour in Rat 253, No. 2, showing small nodule of lightly staining tissue closely resembling normal hyperplastic thyroid. H. & E. × 95.

FIG. 12.—High-power views of section of transplanted $T_{22}$ tumour, showing (*a*) a giant cell and (*b*) an atypical mitosis. H. & E. × 450.

FIG. 13.—Section of lung of rat, killed 6 weeks after inoculation with $T_{22}$ tumour. A metastasis is shown which has penetrated the elastic tissue of the pleura and formed a nodule on the surface of the lung. Gomori elastic tissue stain, H. & E. × 95.

Purves, Griesbach and Kennedy.

Purves, Griesbach and Kennedy.

meet the requirements of the animal. Some residual thyroxine deficiency exists under these conditions, since it is only when the thyrotrophic hormone is unnaturally high that this $T_1$ tumour maintains itself.

*Dependence of the Tumour $T_1$ on thyrotrophic hormone.*

In the second generation, grafting of the tumour experiments were made to determine the conditions necessary for the continued growth of this tumour. Inoculations were made into 4 groups of rats as under :

(*a*) rats on stock diet without special treatment ;
(*b*) rats receiving methylthiouracil in the drinking water ;
(*c*) rats sub-totally thyroidectomized, leaving a small portion of the isthmus in place ;
(*d*) rats totally thyroidectomized.

The results are summarized in Table III. It will be seen that continued growth of the grafts was obtained in the three states of thyroxine deficiency, while no

TABLE III.—*Results of Transplantation Experiments in the Second Generation Transplantation of Tumour $T_1$.*

| Condition of rats. | No. of rats inoculated. | No. of rats with tumour growth. |
|---|---|---|
| Normal . . . . . . . . | 12 | 0 |
| Methyl thiouracil 0·1% in the drinking water | 12 | 10 |
| Subtotally thyroidectomized . . . | 11 | 7 |
| Totally thyroidectomized, . . . . | 13 | 4 |

growth was obtained in the normal animals. This indicates the dependence on high thyrotrophic hormone levels, a dependence which is also displayed when animals bearing growing tumours during the administration of methylthiouracil have their medication stopped. Under these conditions the tumours rapidly regress, and after 6 months the tumours may have entirely disappeared and do not reappear when the animals are again treated with methylthiouracil.

*Structures visible in the fourth generation transplants.*

In the fourth generation transplants of line A (Fig. 1) 3 different types of histological structure were observed, none of them corresponding to the original adenoma.

(1) The bulk of the tumour growing from the graft was a microfollicular adenoma. The acini showed variation in size from minute up to about 15$\mu$ diameter. The large irregularly-shaped acini which were prominent in the $T_1$ tumour were not seen. Colloid was present in some of the acini but was less prominent than in the $T_1$ tumour. The nuclei were hyperchromatic and irregular in shape.

(2) Embedded in this adenomatous tissue was a nodule of tissue distinctly different in staining properties. In this tissue, which had a well-developed acinar structure corresponding to a very hyperplastic normal rat thyroid, the cells were large with abundant cytoplasm. The nuclei were round and did not contain excess chromatin. The lesser numbers of nuclei and their weaker staining properties were responsible for the paler appearance of these nodules in the stained section, causing the nodule to stand out from the group of hyperchromatic nuclei of the adenomatous tissue (Fig. 11). This type of tissue was seen in each of the 2 animals mentioned above and was also seen in one of the grafts of the first generation. The fact that such tissue always forms microscopic nodules and has not ever formed a large part of the grafts in any of the inoculated animals suggests that this type of tissue is a slow-growing variant which arises spontaneously within the adenomatous tissue. The nodules of this type seen in the fourth generation appear to have arisen from the modified adenomatous tissue of these animals mentioned above which differs from that of the original tumour.

(3) The third type of tissue is an anaplastic carcinomatous tissue without any acinar structure. In Animal 253, No. 1, the anaplastic tissue is seen as a discrete mass adjacent to masses of the adenoma but separated from them by fibrous septa. In Animal 253, No. 2, this anaplastic tissue is seen infiltrating and replacing the adenomatous structure without a sharp demarcation (Fig. 8, 10).

*Growth of transplanted Tumour $T_{22}$.*

Tumour tissue from one of the animals (253, No. 1) was minced and injected into 57 young rats which were given 0·01 per cent methylthiouracil. Growth of the transplanted tumour was obtained in 22 of these rats. The growth was much more rapid in this generation than had ever been observed before, tumours of about 1 g. in weight being observed after 10 days. In the fourth week after transplantation animals with the rapidly growing tumours began to succumb. In a proportion of the animals, however, the growth was slower and the animals survived 3 or 4 months. The histology of all these tumours was that of the anaplastic carcinoma described above as the third type of tissue seen in the fourth generation. This tumour, now called $T_{22}$, was successfully maintained by serial transplantation, and in later transplantations successful grafts were obtained in up to 80 per cent of those inoculated. The rate of growth in different animals continued to show a wide range of variation, but was always much more rapid than was obtained with the $T_1$ tumour. The $T_{22}$ tumour has now been transplanted through 7 consecutive generations and has not undergone any progressive change in either malignancy or histology.

*Histology of Tumour $T_{22}$.*

On the cut surface two regions could be regularly seen, the outer with a shiny grey appearance, 5 to 8 mm. broad, and the central one coloured a pale orange with a crumbled surface. Frequently a brown albuminuous fluid had replaced the central tissue. Microscopic examination revealed that, generally, the inner zone of the tumours consisted of degenerating cells or amorphous masses, infiltrated by round cells, and isolated cells with strongly eosinophilic cytoplasm and pycnotic nucleus, lying between the cell debris.

The outer zone, microscopically, appeared similar to that of the anaplastic nodule of the original tumour, and this description applies to both tissues. They consisted of densely crowded cells the majority of which were spindle-shaped, while others were round or of quite irregular shape. Their size varied considerably. There was no proper stroma of the tumour recognizable. Only a few coarse collagen fibres were to be found between the tumour cells. The number of blood-vessels was extremely small. In some parts of the tumour the cells were arranged in strands, with a tendency to form whorls. In other parts of the nodule no special pattern was recognizable. The nuclei also showed considerable variations in shape, size and chromatin content. The smaller elements had dense nuclei, while the larger cells had vesicular nuclei with one, rarely two, rather prominent nucleoli. Unusually large tumour cells were present which had slightly basophilic cytoplasm and nuclei with irregularly dispersed chromatin. Giant cells were present with nuclei varying in number from 2 to 8. The numbers of giant cells found varied somewhat in different animals and were relatively few in the first animal examined. They have, however, been a constant feature of the tumour, and we consider that the tumour should, therefore, be classed as a giant cell carcinoma of the thyroid. Mitotic figures are frequent and many of them atypical.

*Effect of $T_{22}$ tumour on the host.*

The striking feature of the $T_{22}$ tumour is that it kills its host in as short a period as 3 weeks. There is, however, a considerable variation in the rate of growth in different animals, some of them surviving for 2 months or more. In those animals which died within 3 to 4 weeks after inoculation, the animals showed at autopsy an extreme state of emaciation with atrophic muscles and thin soft bones. The tumours' weights varied between 30 and 50 g. After removal of the tumours the carcass weight of the animals was lower than its weight at the time of transplantation. It appears, therefore, that the rapid growth of these tumours withdraws nutrients from the host and leads to the state of emaciation described.

*Effect of thyrotrophic hormone level on Tumour $T_{22}$.*

The $T_{22}$ tumour has been found to grow equally well when inoculated into normal animals or animals receiving methylthiouracil. Moreover, in normal animals in which this tumour is growing, no inhibition of the growth rate has been observed when the animals were treated with thyroxine injections. In some selected animals bearing this tumour in which the growth was relatively slow some were treated with thiouracil. This treatment did not produce any marked acceleration in the growth of these tumours. The $T_{22}$ tumour, therefore, does not show the dependence upon thyrotrophic stimulation which was shown by its parent tumour.

*Iodine metabolism of the $T_{22}$ tumour.*

Rats bearing the $T_{22}$ tumour were injected with radio-active iodine and 24 hours later were killed, and the radio-active iodine concentrations in the tumour tissue and in the blood plasma were measured. The radio-active iodine concentration of the tumour tissue was approximately 50 per cent of that in the plasma. These tumours, therefore, show no selective iodine concentration, a finding which is in agreement with the anaplastic nature of the tumour. Clinical experience shows that iodine concentration is to be expected only in those tumours in which an acinar structure is present.

### DISCUSSION.

*The role of goitrogens in the production of primary tumours in the thyroid.*

When Bielschowsky (1944) described the production of thyroid tumours in the rat by combined treatment with allylthiourea and acetamidofluorene he considered that both the action of the carcinogen plus the hyperplasia resulting from the goitrogen were necessary for the induction of thyroid tumours. However, Griesbach, Kennedy and Purves (1945) reported that the prolonged action of goitrogens alone led to the formation of tumours, which in a later publication (Bielschowsky, Griesbach, Hall, Kennedy and Purves, 1949) were shown to be similar in all respects to those appearing after the carcinogen treatment. With goitrogen alone, however, tumours are later in appearing and are fewer in number. These tumours are dependent upon high thyrotrophic hormone level for their growth. In normal animals such tumours, either in the thyroid or as grafted tumours, undergo rapid regression and eventually disappear, so that they cannot be induced to reappear by the subsequent administration of goitrogen. The simplest explanation, therefore, of the role of the goitrogen in the production of these tumours is that it induces the high thyrotrophic hormone production without which such tumours cannot grow. In this view, the goitrogen would not have any influence on the formation of neoplastic cells. It is, therefore, considered that neoplastic cells arise spontaneously in the normal rat thyroid, and that when conditions are suitable for the growth of such cells, visible neoplasms result. The carcinogen seems to act by speeding up the formation of neoplastic cells. The non-occurrence of thyroid neoplasms in normal rats is explained by the fact that all such primary thyroid tumours in the rat require high thyrotrophic hormone levels for their growth.

*Mutations in transplanted adenomas.*

Since the primary adenomas of the rat thyroid strongly resemble the normal thyroid tissue it is not surprising that there should arise within them areas of tissue differing in structure from the parent adenoma. Presumably these areas arise from changes in single cells of the adenoma similar to the original change which produced the adenoma from the normal thyroid cell. The observation of such changes is facilitated by reason of the large mass of thyroid adenoma that can be maintained, since tumours up to 5 g. in weight are commonly produced. The continuous growth of such tumours and the propagation of them by transplantation leads naturally to the selection of fast-growing types of tissue so that an increase in malignancy with transplantation is to be expected. However, modifications of the adenoma are not invariably in the direction of increased malignancy. We consider that the type of nodule described as the second type of tissue observed in the fourth generation transplants is a relatively slow-growing benign structure which reappears in successive generations, and which by virtue of its slow growth and lack of metastasizing power cannot be selectively propagated by transplantation. On the other hand, the truly malignant, invasive and metastasizing carcinoma will, when it once appears, invariably supplant entirely the benign structure in two, or three at the most, generations of grafting.

*Evolution of malignancy in benign tumours.*

While it has been recognized that transplantable mammalian tumours show increases in malignancy on transplantation, it has been often assumed that such increase in malignancy results from a gradual adaptation to the host or a gradual modification of the original tumour. The well-defined structure of the thyroid adenomata, however, makes it easy to see that modifications of the original tumour arise by distinct mutations and not by gradual changes. Thus, in the parallel line of the $T_1$ tumour strain in which at this time the original structure is still maintained there has been no increase in growth rate or malignant behaviour. Such changes in rate of growth and in malignancy as have been observed in the material at present described have been accompanied by and are due to the formation of a new type of tumour. It is considered that mutations of this sort are of sufficiently frequent occurrence to account for changes in tumour behaviour on transplantation.

It seems important to note that malignant anaplastic tumours similar to the $T_{22}$ have not been seen in over 100 rats which have been examined with primary thyroid tumours. All of these tumours have been of the adenomatous type with well-defined acinar structure. This is true, too, of all the tumours seen after administration of the carcinogen, acetamidofluorene. It therefore seems that the anaplastic tumour is derived from the original thyroid cell by more than one (in this case apparently 3) distinct steps. This observation fits well with the clinical observation that malignant changes in benign tumours are of frequent occurrence, but goes further in suggesting, at least as regards the rat thyroid, malignant tumours can be formed only from benign tumours. It may be found that such a mechanism for the production of malignant tumours may be of more frequent occurrence in human material than is at present realized, since the benign tumour from which the malignant tumour is derived, although it may have existed for a long time, may be quite small and may, therefore, easily escape detection, either at *post mortem* or operation. Furthermore, the benign tumour may not differ very much from the normal tissue and may, therefore, not be recognized as a neoplasm. In this connection it should be noted that while adenomata in the rat thyroid have been observed by many people who have examined rats after long-term goitrogen administration, not all workers have recognized their neoplastic nature.

*Autonomy of thyroid carcinomas.*

It has been considered without the support of experimental evidence that one of the features of neoplasms is that they are not subject to the controlling influences which regulate the growth of normal tissue. However, this hypothesis is one which cannot be tested unless all the influences which regulate growth in normal tissue are first known. Only then is it possible to test experimentally whether the neoplasms are in fact independent of the growth stimuli which normal tissues require. The thyroid adenomata appearing in the rat thyroid are all dependent on thyrotrophic hormone for their existence, and in fact require higher levels of thyrotrophic hormone for their continued growth than does the normal thyroid tissue. Investigation of human thyroid neoplasms with the aid of radio-active iodine have shown that many of these are in fact susceptible to the stimulating influence of thyrotrophic hormone, although neoplasms in human material have not been described which are so entirely dependent upon thyrotrophic hormones as are these primary rat thyroid tumours. The existence of undoubted stimulating effects of thyrotrophic hormone in human thyroid tumours supports the view that these rat thyroid tumours are not exceptional, although their high degree of dependence on thyrotrophic hormone sets them somewhat apart from the thyroid tumours encountered clinically. There seems no reason to hold the view, as some people have done, that these rat tumours should not be classed as neoplasms, since in their case the stimulating hormone or factor on which their growth depends is well characterized and can be artificially manipulated so as to control the tumour growth. Presumably as further knowledge is gained the stimulating influences which condition the growth of other tumours will be discovered, but this should in no way affect their classification as neoplasms.

It is important to note here that from a tumour at first dependent upon a hormonal imbalance for its growth, there has been derived a malignant neoplasm which is no longer dependent upon the stimulating influences which condition the growth of the more benign original tumour. Thus where malignant tumours are found which are not susceptible to any known hormonal influence, the effect of hormonal imbalance in the production of such tumours is not excluded. The existence of hormonal imbalance over a period of years may provide the stimulus to the growth of a primary benign tumour from which a malignant variant is derived, which itself shows no dependence upon hormonal stimulus. This result may have important clinical application if it becomes possible to recognize pre-cancerous states which can be controlled by the variation of hormonal levels, since it suggests a way in which the appearance of malignant tumours might be prevented by appropriate treatment of pre-cancerous states.

### SUMMARY.

The behaviour of a rat thyroid tumour appearing during long-term methyl-thiouracil administration is reported. The tumour was successfully transplanted into rats with thyroxine deficiency. It underwent changes during serial transplantation, three different types of histological structure being produced from the original tumour. One of these structures was a malignant anaplastic carcinoma which was transplantable in rats without thyroxine deficiency.

It is concluded that while primary tumours of the rat thyroid all require high thyrotrophic hormone levels for their growth, tumours of a more malignant character, not influenced by thyrothrophic hormone, may appear by malignant change in the original adenomata. These results have a possible bearing on the prevention of malignancy by the adequate treatment of pre-cancerous states.

### REFERENCES.

BIELSCHOWSKY, F.—(1944) *Brit. J. exp. Path.*, **25**, 90.
*Idem*, GRIESBACH, W. E., HALL, W. H., KENNEDY, T. H., AND PURVES H. D.—(1949) *Brit. J. Cancer*, **3**, 541.
GRIESBACH, W. E., KENNEDY, T. H., AND PURVES, H. D.—(1945) *Brit. J. exp. Path.*, **26**, 18.
PURVES, H. D., AND GRIESBACH, W. E.—(1946) *Ibid.*, **27**, 294.—(1947) *Ibid.*, **28**, 46.